# Medical students’ mental burden and experiences of voluntary work in COVID-19 patient support and treatment services: a qualitative analysis

**DOI:** 10.3205/zma001516

**Published:** 2021-11-15

**Authors:** Christoph Nikendei, Ulrike Dinger-Ehrenthal, Florian Schumacher, Till J. Bugaj, Anna Cranz, Hans-Christoph Friedrich, Sabine C. Herpertz, Valentin Terhoeven

**Affiliations:** 1 Heidelberg University Hospital, Centre for Psychosocial Medicine, Department for General Internal Medicine and Psychosomatics, Heidelberg, Germany; 2 University of Heidelberg, Medical Faculty, Dean's Office, Heidelberg, Germany; 3 Heidelberg University Hospital, Centre for Psychosocial Medicine, Department for General Psychiatry, Heidelberg, Germany

**Keywords:** COVID-19 pandemic, medical education, clinical assignment, psychological support

## Abstract

**Aim: **Medical training is undergoing a dramatic shift toward alternative training methods due to the SARS-CoV-2 pandemic. This study is the first to examine medical students' expectations, experiences, and mental burden related to volunteering in COVID-19 patient support and treatment services using semi-structured interviews.

**Methods:** In May 2020, all 194 Heidelberg University Medical School students involved in volunteer COVID-19 support and treatment services were invited to participate in a cross-sectional, qualitative interview study. The semi-structured interviews were digitally recorded, transcribed, and then analyzed using Mayring's principles for content analysis.

**Results: **We interviewed 12 medical students (8 female, mean age 23.2 years, mean medical training 3.7 years) working in Heidelberg COVID-19 crises management services, i.e., the Heidelberg Medical Hospital COVID-19 inpatient and outpatient units. The analysis revealed two key themes: “Expectations and structural barriers” and “Experiences and mental burden”. The participants reported uncertainty and apprehension before starting their voluntary work. Although they initially found volunteering to be somewhat disorganized, their roles became clearer with time. In addition, they reported good team cohesion, which helped reduce initial concerns and uncertainties. The participants also felt that working in the field had helped them maintain their professional identification while standard medical classes and bedside learning were suspended due to the COVID-19 crises. Overall, they reported little volunteer work-related mental burden.

**Conclusions: ** The participants felt that volunteering during the COVID-19 crisis had benefited their professional development. A designated liaison person, psychosocial support, and introductory and accompanying courses could help alleviate initial concerns and interim difficulties in future crisis-related assignments.

## Zusammenfassung

**Ziel: **Die medizinische Ausbildung erfährt aufgrund der COVID-19-Pandemie eine dramatische Störung, einhergehend mit einer Umstellung auf Online-Unterricht und alternativen Lehrangeboten. Dies ist die erste Studie, die mit Hilfe halbstrukturierter Interviews die Erwartungen, Erfahrungen und psychischen Belastungen von Studierenden bei der freiwilligen Mitarbeit in Betreuungs- und Behandlungsdiensten für COVID-19-Patienten untersucht.

**Methoden:** Im Mai 2020 wurden alle 194 Medizinstudenten, die an der Medizinischen Fakultät der Universität Heidelberg an freiwilligen Unterstützungs- und Behandlungsdiensten, während der COVID-19-Krise beteiligt waren, zur Teilnahme an einer qualitativen Querschnitt-Interview-Studie eingeladen. Die halbstrukturierten Interviews wurden digital aufgezeichnet, wortwörtlich transkribiert und nach den Prinzipien der induktiven Inhaltsanalyse nach Mayring ausgewertet.

**Ergebnisse: **Wir befragten 12 Medizinstudierende (8 weiblich, Durchschnittsalter 23,2 Jahre) mit einer mittleren Ausbildungszeit von 3,7 Jahren, die in verschiedenen COVID-19-Managementdiensten wie der stationären COVID-19-Fachabteilung oder den COVID-19-Ambulanzen der Medizinischen Klinik Heidelberg tätig waren. Die qualitative Analyse ergab zwei zentrale Themen: „Strukturelle Hindernisse und Erwartungen“ und „Erfahrungen“ einschließlich emotionaler Aspekte wie psychische Belastung. Was die strukturellen Hindernisse und Erwartungen betrifft, so erlebten die Studierenden vor Beginn ihrer ehrenamtlichen Tätigkeit Gefühle der Unsicherheit und Befürchtungen. Was die Erfahrungen betrifft, so berichteten die Studierenden von einem Gefühl des Wohlbefindens und der Sicherheit bei der Arbeit in ihren Teams, so dass die eigentliche Arbeit nicht als so einschüchternd oder beängstigend wie befürchtet empfunden wurde. Das klinische Praktikum trug auch dazu bei, die Identifikation der Studierenden mit dem Arztberuf aufrechtzuerhalten, zumal der reguläre Unterricht und das Lernen am Krankenbett während der COVID-19-Krise ausfielen. Insgesamt war die psychische Belastung gering.

**Schlussfolgerungen: **Medizinstudenten sahen ihre klinischen Praktika während der COVID-19-Krise als vorteilhaft für ihre berufliche Entwicklung an. Um anfänglichen Befürchtungen und zwischenzeitlichen Schwierigkeiten entgegenzuwirken, sollten Einführungs- und Begleitkurse, eine zentrale Kontaktperson sowie psychosoziale Unterstützung eingerichtet werden.

## Introduction

Medical training is undergoing a dramatic shift to online and alternative delivery teaching methods due to the COVID-19 pandemic. Consequently, medical students have felt greater stress undergoing medical training under pandemic conditions [1]. The COVID-19 crisis will mark history. The 21^st^ century and, by extension, medical training will be divided into a pre- and a post-COVID-19 era [2]. Around the world, medical schools have suspended clinical internships and bedside teaching in response to the COVID-19 pandemic [3]. As a result, many important learning opportunities will be missed. In addition, medical students face uncertainty about their professional futures, which has been shown to affect their physical and mental well-being negatively [4]. Medical students have already assumed many relief roles in this COVID-19 pandemic: from telephone triaging, remote medical consultations, and child and pet care for health care workers to errand-running, including distributing SARS-CoV-2 safety equipment and coordinating face mask sewing classes while supplies were limited [2], [4], [5]. There is general agreement that medical students have played an essential role in fighting the COVID-19 crisis. In addition, fieldwork is widely recognized as critical to medical training. However, there has been considerable discussion about allowing medical students to work in COVID-19 patient treatment units. Some have argued that medical students are not as yet “essential workers”. Accordingly, medical schools should not expose their protectees to potential physical and mental health hazards [6]. However, research shows that two-thirds of medical students would prefer returning to practical clinical work and learning during the COVID-19 crisis [7] to develop their practical medical skills and professional roles [8]. Therefore, discussions have turned to involve senior medical students in treating COVID-19 patients with the prerequisites of specific training, limited responsibility, and minimal exposure to COVID-19 patients [9], [10].

The present study used qualitative interviews to investigate medical students’ expectations, experiences, and mental burden during their voluntary work in COVID-19 patient support and treatment services. We aimed

1. to explore this stress-prone group’s perceptions of their COVID-19 patient work and
2. to improve our understanding of their experiences and needs to address potential difficulties in future assignments [11].

## Methods

### Study design and ethical considerations

The project was initiated in response to the State Ministry of Baden-Wuerttemberg for Sciences, Research and Arts’ appeal for medical student’s support in health care on 17.03.2020 [https://www.baden-wuerttemberg.de/de/service/presse/pressemitteilung/pid/aufruf-an-studierende-zu-mithilfe-im-gesundheitswesen/]. Before the ministry appeal, the Heidelberg Faculty Council had anticipated that hospital staffing needs would increase dramatically in response to the development of the SARS-CoV-2 pandemic. Hence, after assessing our hospital's short-term staff needs, our dean’s office was tasked with arranging medical student support for health care providers. Following the ministry's appeal, hundreds of medical students volunteered to help during the COVID-19 crisis without clear incentives, i.e., pay or academic credit. Therefore, we must suppose that, at least initially, the medical student volunteers were primarily intrinsically motivated. However, some incentives were created over time: Volunteers were later employed and paid as student assistants (pay grade E3A, about €14 gross/hour) by the Heidelberg University Hospital. In addition, after consultation with the State Examination Office (Landesprüfungsamt), volunteer work was recognized as a clinical elective for regular volunteers while standard medical training was interrupted due to COVID-19 regulations. 

As the crisis was unprecedented and we could only suppose the motives, experiences, and needs of volunteering students, we wanted to research the project systematically. Hence, we decided to use qualitative methods

1. to explore the medical student’s perceptions of and needs during their voluntary work in COVID-19 support and treatment services and
2. to improve our understanding of their support needs (i.e., information, guidance, coping strategies).

Therefore, we conducted a cross-sectional study using qualitative, semi-structured interviews. All interviews were conducted in German. In May 2020, all 194 Heidelberg University Medical Faculty students involved in voluntary support and treatment services during the COVID-19 crises were invited to participate in the interview study per email with the help of the Medical Faculty Dean's Office. Study participation was remunerated with 20 Euros. Our study was approved by the University of Heidelberg ethics committee (S-374/2020), and all participants gave their informed, written consent following the Declaration of Helsinki. 

#### Sample description

At the time of the survey, 194 Heidelberg University Medical Faculty students worked in the Heidelberg University Hospital's COVID-19 support and treatment services. Most medical trainees were deployed in the COVID-19 specialized inpatient intensive care unit (*n*=53), surgical care services (*n*=22), general care service (*n*=18), and emergency ambulance night services (*n*=10). The remaining medical trainees were active in 32 other Heidelberg University Hospital COVID-19 specialized services (*n*=91), including the gastroenterology outpatient clinic, internal medicine care services, Kopfklinik care services, surgical admissions, and the closed psychiatric unit.

#### Semi-structured interviews and qualitative data analysis

The interviews were conducted individually by telephone using a specially developed interview guide (see attachment 1 , Interview guide). The last author conducted and digitally recorded all qualitative interviews. The interviews lasted between nine and 16 minutes. An independent co-worker transcribed the interviews verbatim using Mayring's interview transcription guidelines [12]. Statements were analyzed with the software MAXQDA [13] following Mayring's principles of content analysis [12]. First, sentences were identified in each transcribed interview as quotes, representing most elemental units of meaning [14]. Second, the quotes were coded and summarized into relevant categories and labeled with a short sentence. Then, we checked

1. whether individual text passages fit into one of the categories,
2. whether sentences from the interviews had to be summarized, or
3. whether a new category had to be opened.

In line with our previous work [15], we revised the categories and coded quotes after working through about 40% of the material. Only then did we complete the analysis of the entire material. Next, categories were grouped into main themes until we could define several relevant main themes for all participants. We then discussed our categories and main themes in our research group to reach a consensus and adjusted them if necessary [12]. All interview statements were summarized, and noteworthy differences between the interviews were highlighted. Finally, we included three dichotomous items (yes/no) to assess whether the medical students

1. “Would you volunteer for a COVID-19 assignment again?”,
2. felt well taken care of during the assignment (“Did you feel well taken care of during your assignment?”) and
3. whether they were anxious during their assignment (“Were you anxious during the assignment?”).

Quantitative statistical analysis were carried out with the Statistical Package for the Social Sciences (SPSS) program (Version 26; SPSS Inc.). Demographic data were analyzed using descriptive statistics (frequencies, means, and standard deviations).

## Results

### Sociodemographic characteristics of participating medical students 

The sample characteristics are shown in table 1 (Tab. 1). We interviewed N=12 medical students (8 female) with a mean age of 23.2 years (SD 4.2) and a mean of 3.7 years (SD 1.7) of medical training (range: 1 to 6). The participating medical students were involved in different COVID-19 management settings (see table 1 (Tab. 1)) and volunteered for six weeks on average (range: 3 weeks to 3 months).

#### Main themes

**Figure 1:**
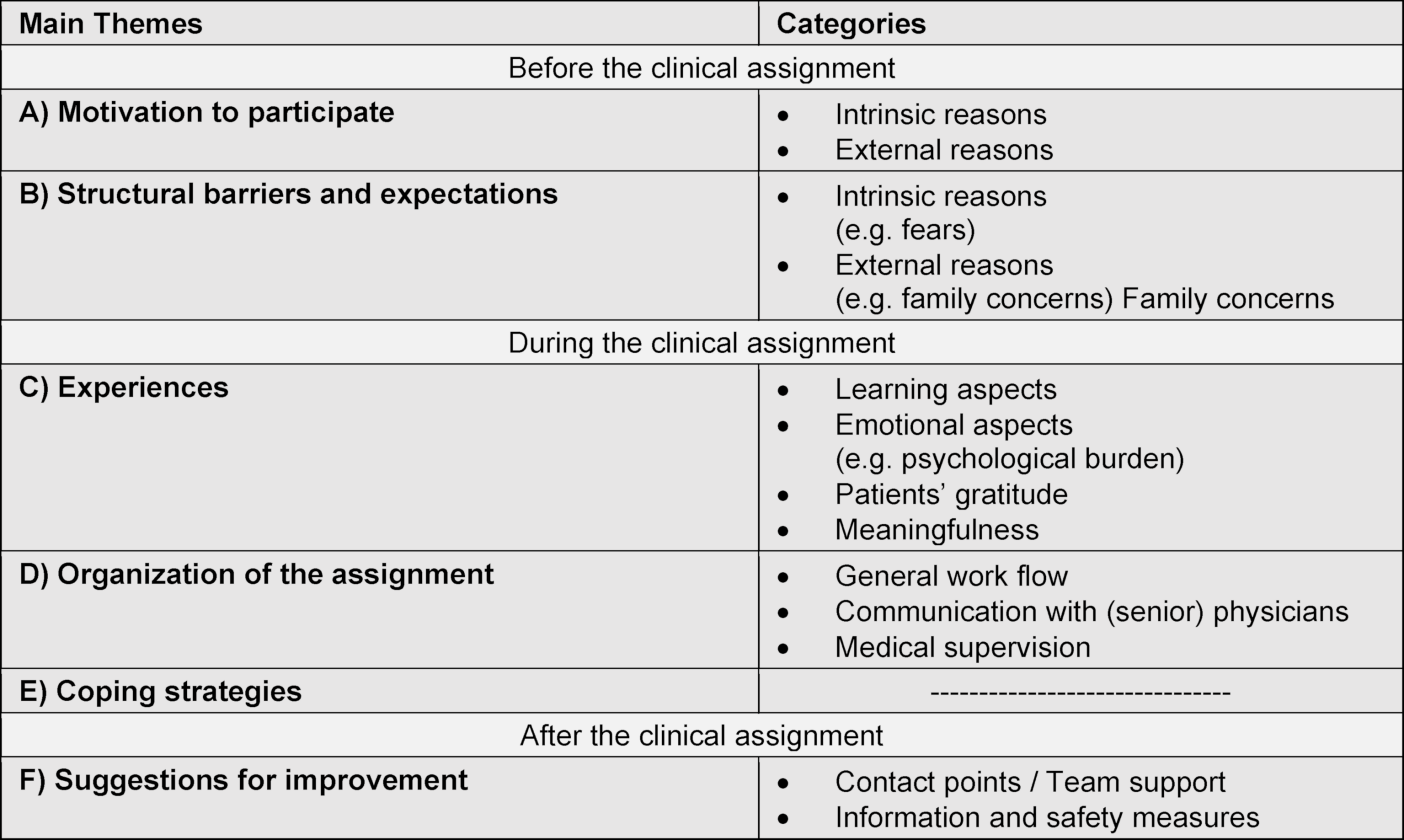
Main themes and categories resulting from qualitative analysis of N = 12 students` semi-structured interviews

We grouped the identified codes [13] into 45 categories (see figure 1 (Fig. 1)), which were summarized into six main themes. For clarity, only the main themes are shown and defined below. Related significant quotations for each category can be found in table 2 (Tab. 2).

#### A. Motivation to volunteer (32 quotations)

The students reported having been motivated to volunteer in the COVID-19 patient treatment and support services by their sense of duty/solidarity. In addition, many said that they wanted to help in the developing crisis. Extrinsic motives primarily included the minister of health’s outreach letter and the deans’ office appeal for help. 

#### B. Structural barriers and expectations (29 quotations)

The students expected working conditions to be chaotic and similar to media reports about the crisis in other countries (i.e., Italy). Personal work overload was a related concern. While participants reported little fear of infection, they did express considerable concern over possibly infecting their families or fellow co-habitants. Volunteering was seen as an opportunity to do something meaningful during the COVID-19 pandemic by helping people. However, the participants’ families were more skeptical and highlighted the increased risk of contracting the disease during volunteer assignments.

#### C. Experiences (62 quotations)

The participants reported that the first few shifts were unorganized and stressful because general routines and the individual workflows had not been established yet. As teams had been thrown together quickly, it took time for everyone to find their respective roles. However, the participants reported that routines were soon established. Many participants felt that, over time, the volunteer assignment felt like a regular clinical internship and did not cause extraordinary levels of stress or concern. 

Most participants felt that their volunteer work was a rewarding experience. The fieldwork had increased their professional and academic motivation. The participants highlighted that they had learned many practical clinical skills, made professional connections, and developed personally. Participants felt that working in the field had also improved their communication skills. In addition, they were encouraged by seeing their patients’ condition improve, and named their patients’ gratitude was particularly rewarding. Furthermore, the participants cherished the commitment, support, and encouragement from their team members. Most said they had generally enjoyed working in the COVID-19 support services and had been shown much gratitude by their patients and teams. Overall, participants agreed that the positive experiences had outweighed the negative ones during their assignments. In addition, the participants felt that their deployment had given them the opportunity for personal and professional growth by allowing them to experience firsthand that they could navigate under challenging conditions.

The participants’ perceptions differed considerably concerning their volunteer work-related mental burden. While one participant thoroughly enjoyed the experience, others did report feeling burdened at times. For example, some participants said they had sometimes felt preoccupied after their shifts and were more mindful of the fact that the pandemic was still developing. Still, others reported that the first few days of seeing COVID-19 patients arrive had been quite harrowing for them. One participant, in particular, shared their sense of feeling overwhelmed by the situation. In sum, however, the participants generally reported little mental burden related to their volunteer assignment. Nevertheless, the participants were very concerned about possibly infecting family and friends.

Most students experienced a great sense of purpose in their COVID-19 volunteer work: While they felt active in tackling the crisis, their work was also highly appreciated. The participants believed that they had learned a great deal by working in the field during this crisis. Likewise, the students were glad to have something to do during the pandemic since they could not go about their regular routines due to the COVID-19 Lockdown regulations.

#### D. Organization of the assignment (38 quotations)

The participants criticized that volunteer work was initially unstructured and tasks were unclear. However, generally, administrative matters had improved with time. By contrast, participants felt that their units' good team cohesion and group communication had been supportive. Similarly, medical treatment-related supervision by senior team members was also appreciated.

#### E. Coping strategies (13 quotations)

The participants reported dealing with their assignment-related worries by talking to others. Some participants felt burdened after their shifts. However, nearly all participants thought they could speak to their friends, flatmates, or family about their concerns which helped them. In addition, one participant said that they self-soothed by engaging in mindfulness-based internal dialogue. The other participants mentioned no other specific strategies related to dealing with worries.

#### F. Suggestions for improvement (18 quotations)

The participants suggested that a liaison person or psychosocial support services for COVID-19 associated workers would be advisable in future crisis assignments. In addition, they suggested providing volunteers with more detailed information on necessary medical clinical procedural and communication skills, i.e., instructional videos or written/ illustrated instructions. The participants felt that easy access to information would help address initial concerns and insecurities and give volunteering medical students more orientation, especially at the beginning of their assignments. Furthermore, some participants wanted to see regular COVID-19 testing for volunteers to help protect their families and friends. 

#### Quantitative analysis of dichotomous questions

n=11 participants responded that they would volunteer again. However, while n=9 felt well taken care of during their assignment, only n=6 respondents answered feeling no concern regarding their COVID-19-related volunteer work (e.g., fear of infection or infecting someone).

## Discussion

The current study assessed medical students’ expectations, experiences, and mental burden after volunteering in COVID-19 patient support and treatment while traditional teaching formats were suspended during the COVID-19 crisis. The COVID-19 pandemic has undoubtedly necessitated a catalytic increase in digital/hybrid and alternative teaching concepts [16]. As research in this area has flourished, discussions regarding the pros and cons have also become more lively. However, although many learning opportunities are missed in digital formats, others are gained: For example, Rahm et al. (2021) examined the effects of real e-learning cases on students' learning motivation during the COVID-19 pandemic. They could show that working with e-learning patient cases motivated students and helped mitigate the lack of face-to-face, bedside teaching during the COVID-19 pandemic [17]. Similarly, others have concluded that distance learning might not only be “better than nothing”, but can be hugely beneficial under challenging circumstances. For instance, all participants responded well to switching essential exam preparation courses to digital teaching formats [18]. With the global climate crisis in mind, yet another benefit of digitalization worth noting lies in the reduction of the scientific community’s impact on the climate through lower resource consumption and less commuting [19].

Nevertheless, it is undisputed that workplace learning is still critical in medical training [20], as outlined in the CANMEDS training framework [21], which describes physician skill development. Here, direct bedside teaching is highlighted as critical to developing future physicians’ practical clinical skills and ensuring high-quality, safe, patient-centered care [22]. 

Our results suggest that COVID-19 volunteer clinical assignments were valuable for developing the participants’ practical-medical and communication skills. The participants saw their volunteer work as a meaningful opportunity that helped them build their clinical experience. Consistent with Mühlbauer et al.’s (2021) quantitative examination of n=244 medical students’ participation in COVID-19 care, the interviewees were strongly motivated to volunteer because they wanted to do something meaningful and helpful during the pandemic [23]. Most interviewed medical students said they would volunteer in COVID-19 crisis services again. However, they did feel uncertainty and apprehension before starting their work in COVID-19 settings. Some anticipated disorganization and feared being overburdened by the workload and the patients’ emotional and physical burden. Similarly, Weurlander et al. (2019) could show that medical students often felt uncertainty before imminent emotional challenges [24]. While the participants experienced initial disorganization and uncertainty in the COVID-19 units, the haphazardly thrown-together team soon developed a strong cohesion. Hence, all interviewed medical students enjoyed working in the COVID-19 services and felt supported and appreciated by their teams and patients. The initial fears and concerns were therefore not realized. In part, the favorable circumstance that only a comparatively small number of COVID patients were being treated at Heidelberg University Hospital at the beginning of 2021 may have contributed to its positive outlook. Moreover, few severe disease outcomes occurred at the time. Interestingly, our results are similar to the expectations and experiences of medical students involved in establishing medical help services during the 2015 refugee crisis in Germany [25], [26]. Overall, the participants felt comfortable in their volunteer assignments and had access to valuable learning opportunities at a time when standard medical training was suspended in Germany. The volunteer work also helped the interviewed students retain their identification with the medical profession while distance-learning formats were still being established [24]. Many students were concerned that their academic futures might be in jeopardy because regular teaching and bedside learning had been suspended, and libraries had been closed during the COVID-19 crises [27]. Furthermore, some participants were concerned that although they were now involved in treating COVID-19 patients, it was unclear whether they could continue their work due to developing German COVID-19 quarantine and lockdown regulations. Hence, while medical supervision was perceived as helpful, the interviewees felt that an external designated liaison person might benefit volunteer medical students in the future. 

Overall, the participants felt well taken care of during their COVID-19 clinical assignment and did not experience much physical or mental burden. Nonetheless, it must be noted that the COVID-19 situation was largely controlled at the time in Germany, with only a few severe cases being treated in the hospital at the time. Furthermore, at the time, violent outbreaks with chaotic conditions, such as those previously seen in the media in Italy or India, did not occur in Germany until early 2021. However, considering that the initial COVID-19 wave was mild in Germany relative to other countries, our data suggest that volunteer medical students may require mental support while working with severely ill COVID-19 patients in crisis conditions. We are sure that a more substantial infection wave will cause significantly more mental burden in people working in COVID-19 specialized units. However, it is reasonable to assume that the sample in our study is an exceptionally resilient group, even for a stress-tested population of medical students [11], and can not be generalized. 

This study also explored the interviewed medical students’ perceived needs related to mental burden and their coping methods for COVID-19 volunteer work-related burden. While most participants confided in their social networks for emotional support, all would have liked to have known more about their tasks were going to beforehand. Particularly at the outset of their COVID-19 assignments, they would have liked to have access to low-threshold informational materials, such as task guidelines or information videos. Many felt that their initial uncertainties and fears could be addressed by more extensive preparation before the actual COVID-19 assignments. Moreover, an accompanying COVID-19 psychosocial support program involving self-reflection and regular supervision with a senior professional would also be desirable. In general, medical supervision is characterized by experienced physicians teaching students medical knowledge and skills (educational dimension) and providing them with the opportunity to talk about stressful experiences at work (supportive psychological dimension) and administrative aspects. Medical supervision, therefore, ensures the safety of patients and trainees [28]. The dynamic COVID-19 developments in Germany in the winter of 2020 especially have further underscored the need for such support programs.

Studies have shown that medical students can experience secondary traumatization during their practical clinical training when working with survivors of traumatic events [29]. In light of the current pandemic dynamics, this phenomenon could also occur among health care workers treating critically ill COVID-19 patients [30]. Therefore, special training on coping with extreme situations, such as a pandemic, could safeguard students’ mental health in the future and should be considered in standard teaching practice [29]. On the other hand, studies have shown that students working with refugees show few signs of secondary traumatic stress [25]. So far, our data suggest that medical students working with COVID-19 patients also show few signs of mental burden. Nevertheless, considering that our data was gathered during a mild COVID-19 wave, an introductory workshop, a designated liaison person, and psychosocial support should be established to address initial fears in future crisis assignments. 

### Limitations

Due to the small sample size, the power of the study is limited and may not be representative of the overall sample population. However, content saturation was reached, as proposed for qualitative studies. Likewise, there are limitations to the generalizability of our data because the infectious situation varies widely from site to site.

## Conclusions

The current study sheds light on medical students' experience of COVID-19 related volunteer clinical assignments. Furthermore, it underlines the areas where psychological support and medical preparation can be improved. We recommend establishing support for medical students working in COVID-19 settings. Introductory workshops, a liaison person, and psychosocial support can help address initial and developing concerns and alleviate intermediate difficulties in future crisis assignments. In sum, engaging medical students’ in voluntary support of COVID-19 patients seems like an excellent way to promote individual growth, identification with the medical profession, and bridge distance learning gaps in medical training. However, medical students need to be well prepared and supported by their assigned units during their volunteer work in this potentially stressful setting.

## Competing interests

The authors declare that they have no competing interests. 

## Supplementary Material

Interview guide

## Figures and Tables

**Table 1:**
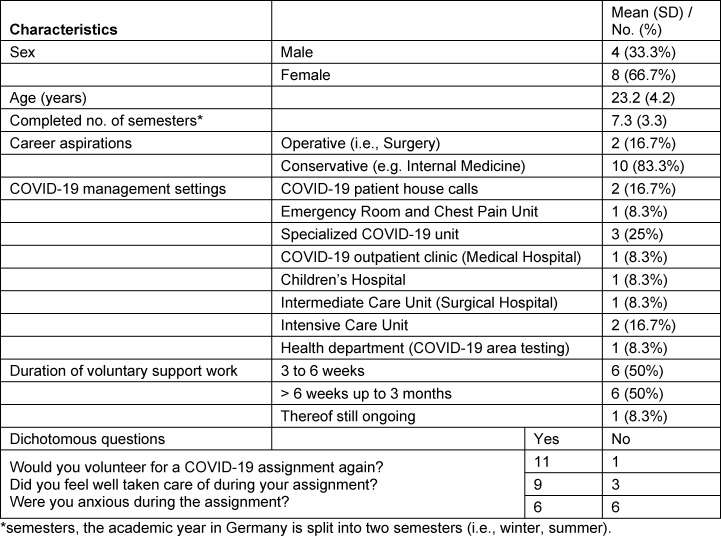
The study sample's sociodemographic characteristics (N=12)

**Table 2:**
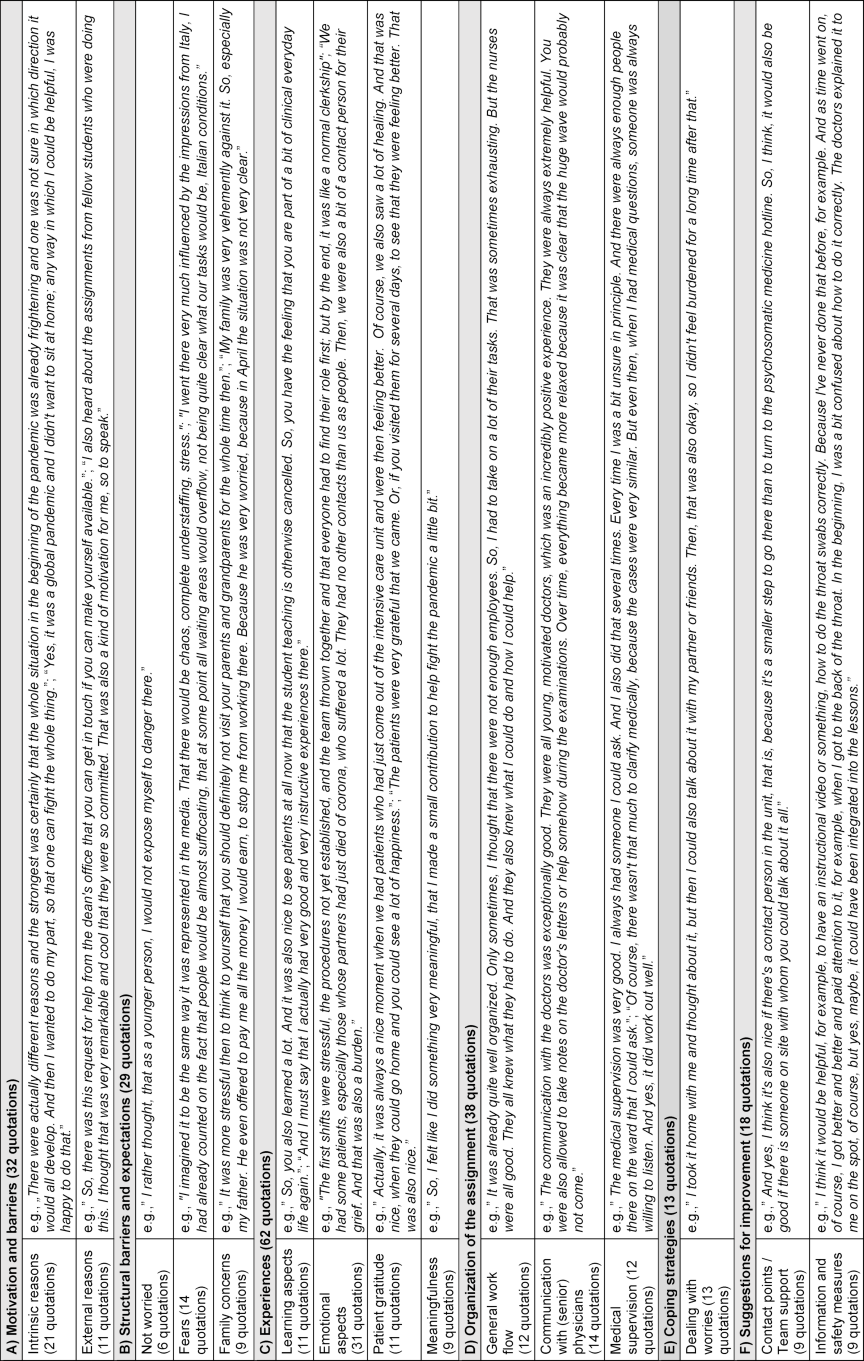
Main themes and related significant quotations of the categories

## References

[1] Loda T, Loffler T, Erschens R, Zipfel S, Herrmann-Werner A (2020). Medical education in times of COVID-19: German students' expectations - A cross-sectional study. PLoS One.

[2] Chinelatto LA, Costa TRD, Medeiros VMB, Boog GHP, Hojaij FC, Tempski PZ, de Arruda Martins M (2020). What You Gain and What You Lose in COVID-19: Perception of Medical Students on their Education. Clinics (Sao Paulo).

[3] Li HO, Bailey AM (2020). Medical Education Amid the COVID-19 Pandemic: New Perspectives for the Future. Acad Med.

[4] Chandratre S (2020). Medical Students and COVID-19: Challenges and Supportive Strategies. J Med Educ Curric Dev.

[5] Kratochvil TJ, Khazanchi R, Sass RM, Caverzagie KJ (2020). Aligning student-led initiatives and Incident Command System resources in a pandemic. Med Educ.

[6] Menon A, Klein EJ, Kollars K, Kleinhenz AL (2020). Medical Students Are Not Essential Workers: Examining Institutional Responsibility During the COVID-19 Pandemic. Acad Med.

[7] Compton S, Sarraf-Yazdi S, Rustandy F, Kumar Radha Krishna L (2020). Medical students' preference for returning to the clinical setting during the COVID-19 pandemic. Med Educ.

[8] Sierpina VS (2020). The impact of COVID-19 on medical education. Explore (NY).

[9] Wang JH, Tan S, Raubenheimer K (2020). Rethinking the role of senior medical students in the COVID-19 response. Med J Aust.

[10] Wendel Garcia PD, Massarotto P, Auinger K, Schuepbach RA, Klinzing S (2020). Students Supporting Critical Care - A contention plan to prevent the decompensation of ICUs in the COVID-19 pandemic:Translating Bjorn Ibsens' polio-lessons to modern times. Crit Care.

[11] Erschens R, Keifenheim KE, Herrmann-Werner A, Loda T, Schwille-Kiuntke J, Bugaj TJ, Nikendei C, Huhn D, Zipfel S, Junne F (2019). Professional burnout among medical students: Systematic literature review and meta-analysis. Med Teach.

[12] Mayring P (2010). Qualitative Inhaltsanalyse. Grundlagen und Techniken.

[13] MAXQDA P (1989). MAXQDA, software for qualitative data analysis (Version 10).

[14] Corbin J, Strauss A (2014). Basics of qualitative research: Techniques and procedures for developing grounded theory.

[15] Zehetmair C, Nagy E, Leetz C, Cranz A, Kindermann D, Reddemann L, Nikendei C (2020). Self-Practice of Stabilizing and Guided Imagery Techniques for Traumatized Refugees via Digital Audio Files: Qualitative Study. J Med Internet Res.

[16] Weissmann Y, Useini M, Goldhahn J (2021). COVID-19 as a chance for hybrid teaching concepts. GMS J Med Educ.

[17] Rahm AK, Tollner M, Hubert MO, Klein K, Wehling C, Sauer T, Hennemann HM, Hein S, Kender Z, Günther J, Wagenlechner P, Bugaj TJ, Boldt S, Nikendei C, Schultz JH (2021). Effects of realistic e-learning cases on students' learning motivation during COVID-19. PLoS One.

[18] Anschuetz W, Wagner F, Jucker-Kupper P, Huwendiek S (2021). Workshops for developing written exam questions go online: appropriate format according to the participants. GMS J Med Educ.

[19] Nikendei C, Cranz A, Bugaj TJ (2021). Medical education and the COVID-19 pandemic - a dress rehearsal for the "climate pandemic"?. GMS J Med Educ.

[20] Bugaj TJ, Schmid C, Koechel A, Stiepak J, Groener JB, Herzog W, Nikendei C (2017). Shedding light into the black box: A prospective longitudinal study identifying the CanMEDS roles of final year medical students' on-ward activities. Med Teach.

[21] Frank JR, Danoff D (2007). The CanMEDS initiative: implementing an outcomes-based framework of physician competencies. Med Teach.

[22] Frank JR, Snell L, Sherbino J, Boucher A (2015). CanMEDS 2015 Physician Competency Framework.

[23] Mühlbauer L, Huber J, Fischer MR, Berberat PO, Gartmeier M (2021). Medical students' engagement in the context of the SARS-CoV-2 pandemic: The influence of psychological factors on readiness to volunteer. GMS J Med Educ.

[24] Weurlander M, Lonn A, Seeberger A, Hult H, Thornberg R, Wernerson A (2019). Emotional challenges of medical students generate feelings of uncertainty. Med Educ.

[25] Kindermann D, Jenne MP, Schmid C, Bozorgmehr K, Wahedi K, Junne F, Szecsenyi J, Herzog W, Nikendei C (2019). Motives, experiences and psychological strain in medical students engaged in refugee care in a reception center- a mixed-methods approach. BMC Med Educ.

[26] Kindermann D, Schmid C, Derreza-Greeven C, Junne F, Friederich HC, Nikendei C (2019). Medical Clerkship in a State Registration and Reception Center for Forced Migrants in Germany: Students' Experiences, Teachable Moments, and Psychological Burden. Int J Environ Res Public Health.

[27] Papapanou M, Routsi E, Tsamakis K, Fotis L, Marinos G, Lidoriki I, Karamanou M, Papaioannou TG, Tsiptsios D, Smyrnis N, Rizos E, Schizas D (2021). Medical education challenges and innovations during COVID-19 pandemic. Postgrad Med J.

[28] Kilminster SM, Jolly BC (2000). Effective supervision in clinical practice settings: a literature review. Med Educ.

[29] Crumpei I, Dafinoiu I (2012). Secondary traumatic stress in medical students. Procedia Soc Behav Sci.

[30] Arpacioglu S, Gurler M, Cakiroglu S (2021). Secondary Traumatization Outcomes and Associated Factors Among the Health Care Workers Exposed to the COVID-19. Int J Soc Psychiatry.

