# Supplementary material for: Medical students’ mental burden and experiences of voluntary work in COVID-19 patient support and treatment services: a qualitative analysis
Source: GMS J Med Educ. 2021 Nov 15;38(7):Doc120. doi: 10.3205/zma001516 (PMC8675374; doi:10.3205/zma001516)
Supplement: Interview guide [file JME-38-7-120-s-001.pdf]

Attachment 1: Interview guide

Information to the study participant
Dear Mr./Mrs...as you know, we want to explore the personal experiences of students involved in the care of COVID-19 patients during this phone call interview. We hope that your answers will help us better understand encountered challenges and help support and improve people's mental health in COVID-19 patient care. In addition, by agreeing to participate in our study, your input will help us provide students working with COVID-19 patients with better need-informed information, interventions, and professional support in the future. Thank you very much for your support in our research efforts!<br><br>You are currently involved in the care of COVID-19 patients. We would now like to ask you a few questions about your work. Our interview will last about 20 minutes. Feel free to answer the questions in any way you like. In addition, I might ask you some additional questions in response to your answers to clarify them. Do you have any questions?

Question 1: Motivations of use		
**Guiding question**	**Maintaining questions**	**Additional questions**
To begin with, I would be interested to know what prompted you to volunteer to help care for the COVID-19 patients?	Personal motivation?<br>External cause?<br>Support?<br>Discussion with others?<br>Etc.	-

Question 2: Anticipated charges		
**Guiding question**	**Maintaining questions**	**Additional questions**
What do you think working with COVID-19 patients would be like before your assignment?	What fears or concerns did you have before your first assignment?<br>What did you think contact with COVID-19-tested individuals would be like before your first assignment?	-

Attachment 1 to: Nikendei C, Dinger-Ehrenthal U, Schumacher F, Bugaj TJ, Cranz A, Friedrich HC, Herpertz SC, Terhoeven V. *Medical students‘ mental burden and experiences of voluntary work in COVID-19 patient support and treatment services: a qualitative analysis.* GMS J Med Educ. 2021;38(7):Doc120. DOI: 10.3205/zma001516

Question 3: Subjective experience of the assignment		
**Guiding question**	**Maintaining questions**	**Additional questions**
How did you experience working in COVID-19 patient care?	Did your impressions change over time?<br>-What associations did you have?<br>How did you experience contact with COVID-19-tested individuals in particular?<br>-What feelings did you have while working with COVID-19-tested individuals?<br>-Were there situations you found challenging?<br>-Did you experience any good moments?	Was it depressing?<br>Did it make you feel anxious or fearful?<br>Did you feel panicked yourself?<br>Did you feel powerless?<br>Or did you not "feel" much at all?
	What do you think about the level of organization of your assignment?<br><br>What was the level of communication within the team and with the physicians?<br><br>How did you experience the available supervision on a medical level?	Did you feel well taken care of during your assignment?<br>Were you able to consult experienced physicians when making difficult medical decisions?

Question 4: Traumatization		
**Guiding question**	**Maintaining questions**	**Additional questions**
People working in close contact with physically and mentally burdened patients, for example, because of uncertainties surrounding the disease and its progression, may also find that they become mentally burdened.<br>Would you say that you have experienced something like this yourself?	(Explore depression and anxiety symptoms)	-

Question 5: Subjective experience after deployment		
**Guiding question**	**Maintaining questions**	**Additional questions**
After caring for COVID-19 patients, did you ever feel preoccupied with something related to your work there?	What thoughts or feelings were on your mind when you went home in the evening?<br>Has anything related to your assignment kept you preoccupied in particular?<br>How do you deal with your experiences during your assignment?<br>Have you talked to anyone?<br>Have you asked for support? Etc.	-

Question 6: Subjective experience after deployment		
**Guiding question**	**Maintaining questions**	**Additional questions**
Do you think that your values or outlook have changed after working on this assignment?	-	-

Question 7: Interventions?		
**Guiding question**	**Maintaining questions**	**Additional questions**
What kind of support do you think students working on this assignment need?	What would be helpful from a medical perspective?<br>What would be helpful for your mental health?	-

Question 8: Further assignments?		
**Guiding question**	**Maintaining questions**	**Additional questions**
Would you volunteer for an assignment working with COVID-19 patients again?	-	-

Question 9: Conclusion		
**Guiding question**	**Maintaining questions**	**Additional questions**
In conclusion, what have you been able to draw from your experience caring for COVID-19 patients in the field?	-	-
